# Identification and validation of a cancer-associated fibroblasts-related scoring system to predict prognosis and immune landscape in hepatocellular carcinoma through integrated analysis of single-cell and bulk RNA-sequencing

**DOI:** 10.18632/aging.205099

**Published:** 2023-10-18

**Authors:** Lingling Bao, Xuede Zhang, Wenjuan Wang, Bitao Jiang

**Affiliations:** 1 Department of Hematology and Oncology, Beilun District People’s Hospital, Ningbo, China; 2 Department of Oncology, Weifang People’s Hospital, Weifang, China

**Keywords:** cancer-associated fibroblasts, prognosis, immune microenvironment, biomarker, hepatocellular carcinoma

## Abstract

Background: Cancer-associated fibroblasts (CAFs) regulate the malignant biological behaviour of hepatocellular carcinoma (HCC) as a significant component of the tumour immune microenvironment (TIME). This study aimed to develop a CAFs-based scoring system to predict the prognosis and TIME of patients with HCC.

Methods: Data for the TCGA-LIHC and GSE14520 cohorts were downloaded from The Cancer Genome Atlas and the Gene Expression Omnibus databases. Single-cell RNA-sequencing data for HCC samples were retrieved from the GSE166635 cohort. The Least Absolute Shrinkage and Selection Operator algorithm was employed to develop a CAFs-related scoring system (CAFRss). The predictive value of the CAFRss was determined using Kaplan-Meier, Cox regression and Receiver Operating Characteristic curves. Additionally, the TIMER platform, single sample Gene Set Enrichment Analysis and the Estimation of STromal and Immune cells in MAlignant Tumour tissues using Expression data algorithms were performed to determine the TIME landscape. Finally, the pRRophic algorithm was utilised for drug sensitivity analysis.

Results: The evaluation of the CAFRss system demonstrated its superior ability to predict the clinical outcome of patients with HCC. Additionally, CAFRss effectively distinguished HCC populations with distinct TIME landscapes. Furthermore, CAFRss-based risk stratification identified individuals with immune ‘hot tumours’ and predicted the survival of patients treated with ICBs.

Conclusions: The developed CAFRss can serve as a predictive tool for determining the clinical outcome of HCC and differentiating populations with diverse TIME characteristics.

## INTRODUCTION

Primary liver cancer (PLC) is currently one of the most prevalent malignancies of the digestive system. Global Cancer Statistics reports that PLC ranks sixth in incidence and third in mortality among malignancies worldwide [1]. Hepatocellular carcinoma (HCC) accounts for approximately 80% of all PLC cases, and is the predominant subtype of liver cancer [2, 3]. Although targeted drugs, such as tyrosine kinase inhibitors (TKIs) and combination therapies, have improved survival outcomes of patients with HCC, the heterogenous nature of HCC significantly impacts treatment efficacy and patient prognosis [4, 5]. Hence, there is an urgent need to identify specific biomarkers to determine the clinical outcomes and guide personalised therapeutic approaches for patients with HCC.

The interaction between tumour cells and the tumour microenvironment (TME) plays a crucial role in tumourigenesis, progression, metastasis and treatment [6, 7]. Within the TME, cancer-associated fibroblasts (CAFs) are key components that influence malignant behaviours, such as tumour invasion, metastasis, immune escape and drug resistance [8–11]. Studies have demonstrated that CAFs contribute to the progression of HCC by altering tumour cell stemness, promoting metabolic reprogramming and enhancing tumour angiogenesis [12–15]. Additionally, CAFs shape the suppressive tumour immune microenvironment (TIME) in HCC by secreting macrophage migration suppressors [16]. Furthermore, CAFs modulate various immune cells in the TIME of HCC, either directly or through secreted components [17]. Therefore, exploring the biomarkers related to CAFs is vital for predicting the prognosis of HCC and assessing the diversity of TIME.

In the present study, we developed a CAFs-related scoring system (CAFRss) using single-cell sequencing and The Least Absolute Shrinkage and Selection Operator (LASSO) algorithm. The system demonstrates superior predictability for the clinical outcome of patients with HCC. Additionally, CAFRss effectively characterises the TIME profile, enabling the differentiation between ‘hot’ and ‘cold’ immune tumours and guiding the use of immune checkpoint blockades (ICBs). These findings provide a basis for selecting individualised therapeutic regimens for patients with advanced HCC.

## MATERIALS AND METHODS

### Data collection

**Figure 1:**
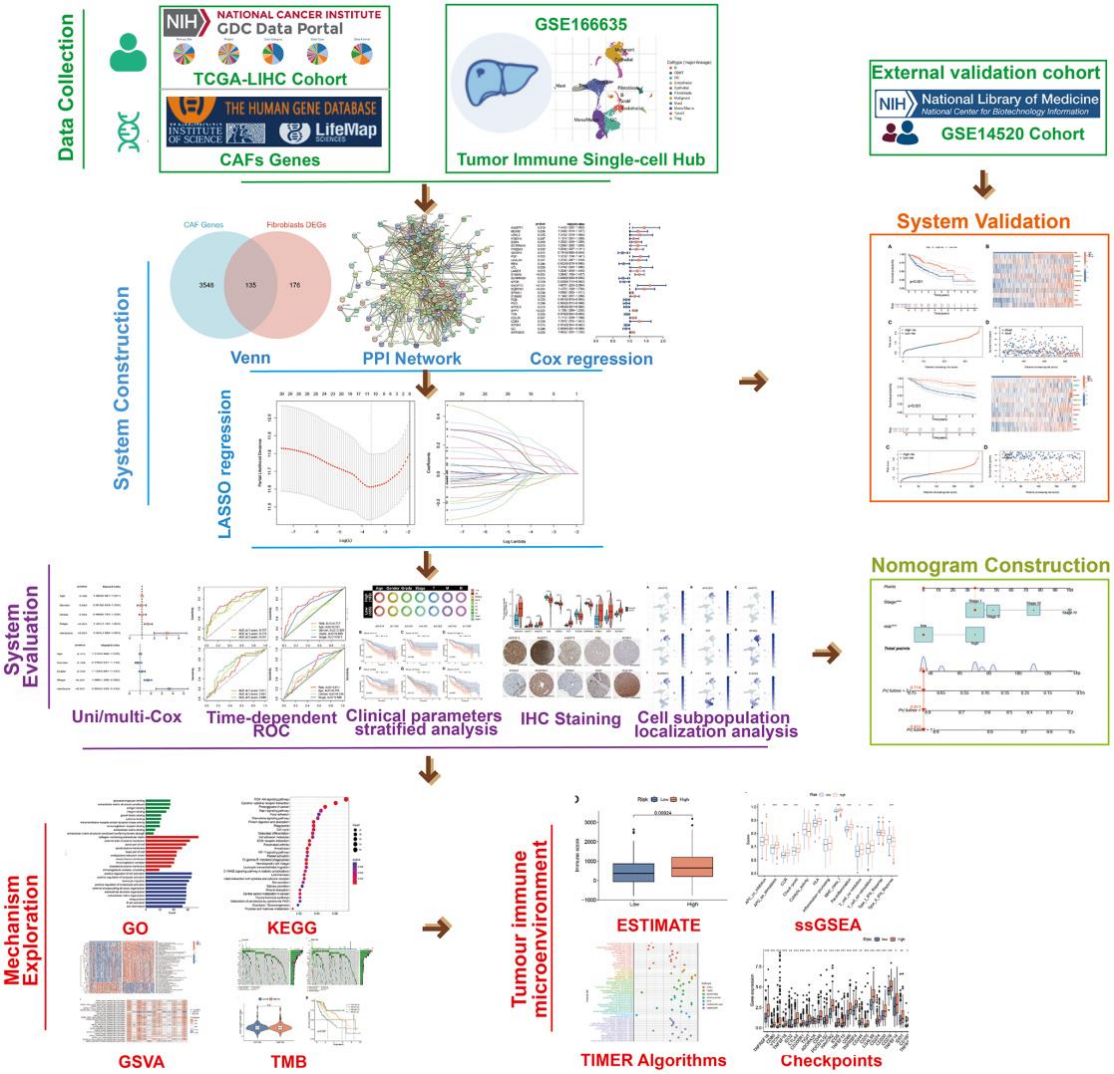
The flow chart of the study.

The flow chart of the study is presented in Figure 1. The transcriptomic and clinical data for individuals with HCC were downloaded from The Cancer Genome Atlas (TCGA) (https://portal.gdc.cancer.gov/repository) repository. The nucleotide variation matrix downloaded from the TCGA-LIHC cohort was collated using Perl scripts (V.5.32.1.1) to generate the tumour mutation burden (TMB) matrix for subsequent analysis. The transcriptomic and clinical parameter data for the HCC cohort GSE14520 were downloaded from the Gene Expression Omnibus (GEO) database (https://www.ncbi.nlm.nih.gov/). Single-cell sequencing data for the GSE166635 cohort were obtained from the Tumor Immune Single-cell Hub (TISCH) database (http://tisch.comp-genomics.org/), wherein annotations referring to the major-lineage entry in the TISCH and the existing classical cell markers were made. Immunohistochemical (IHC) staining images of proteins encoded by CAFRss-related genes *ANGPT1*, *IGFBP4*, *S100A9*, *SERPING1*, *ANGPT2*, *SQSTM1*, *SPINK1*, *FGB*, *SPP1* and *AKR1B10* in HCC tumour tissue were obtained from the Human Protein Atlas V.22.0 (HPA) (https://www.proteinatlas.org) (Supplementary Table 1) [18]. Finally, the set of CAFs-associated genes were obtained from The Human Gene Database (https://www.genecards.org/) [19].

### Identification of differentially expressed genes (DEGs) between CAFs and other cells

DEGs (fold change >1.5, *P* < 0.05) between CAFs and other cell types were extracted from single-cell sequencing data from the GSE166635 cohort. Furthermore, the R package ‘VennDiagram’ was used to draw a Venn diagram of DEGs and CAFs-associated genes to identify overlapping CAFs-associated genes. Protein interactions of the intersecting genes were mapped using the STRING platform (https://cn.string-db.org/).

### Development of a CAFRss for HCC

CAFs-related genes associated with survival in the TCGA-LIHC cohort were identified using a univariate (uni-) Cox regression algorithm (*P* < 0.05). To avoid overfitting, the LASSO regression algorithm was employed to select the relevant genes for developing the scoring system. The risk scores for patients with HCC were calculated based on the risk coefficients of the selected genes using the following equation:


$$\text{Risk}\;\text{score}\;(\text{CAFRss})\;=\;\sum_{i=1}^{n}\text{Coefficient}(i)\;\times \;\text{Expression}(i)$$

Risk stratification was performed for all patients with HCC based on the median risk score of individuals in the TCGA-LIHC cohort.

### Verification and assessment of the CAFRss

Survival validation was conducted using Kaplan-Meier (K-M) curves for patients in the TCGA-LIHC and GSE14520 cohorts, utilising the ‘survivor’ and ‘survminer’ packages. The ‘pheatmap’ was utilised to generate the expression status graph of genes in the scoring system. Uni- and multivariate (multi-) Cox regression analyses were performed to assess the independent prognostic ability of the CAFRss. Additionally, the predictive performance of the CAFRss was analysed using the ‘ggplot2’, ‘grid’, ‘RcolorBrewer’, ‘reshape2’ and ‘tidyverse’ R packages, wherein Receiver Operating Characteristic (ROC) curves were drawn and compared with other clinicopathological parameters. Furthermore, K-M curves were plotted for different stratified subgroups based on various clinical parameters to evaluate the applicability of the CAFRss.

### CAFRss-based nomogram construction

Cox regression identified CAFRss and TMN stage as independent prognostic predictors of HCC. These factors were incorporated into the construction of the prediction nomogram to enhance the prediction of patient prognosis. The predictive accuracy of the nomogram was evaluated using calibration curves generated by the Hosmer–Lemeshow test (method = ‘boot’). The nomogram was constructed with the packages ‘survival’, ‘regplot’ and ‘rms’.

### CAFRss-based functional enrichment analysis

Gene Set Variation Analysis (GSVA) was performed using the ‘limma’, ‘GSVA’, ‘reshape2’, ‘GSEABase’, ‘pheatmap’ and ‘ggplot2’ packages to determine the the enrichment of the Kyoto Encylopaedia of Genes and Genomes (KEGG) pathways in different risk subgroups [20]. Additionally, we analysed the correlation between CAFRss-related gene expression and tumour-associated signalling pathways, constructing a correlation heat map.

DEGs between the two risk groups were determined using the ‘limma’ (fold change >2). Gene Ontology (GO) analyses were performed to explore the extent of enrichment of DEGs in biological processes, molecular function and cellular components. Furthermore, KEGG analysis was also performed to investigate the enrichment of DEGs between different signalling pathways.

### Correlation analysis of the CAFRss with TMB

Simple nucleotide variation data from the TCGA cohort were collated and processed using Perl to generate a TMB matrix. The ‘limma’ and ‘ggpubr’ software packages were employed to compare and visualize the difference in TMB between the two risk subgroups. K-M survival analysis explored survival differences between different TMB subgroups and combinations of risk subgroups. Additionally, the ‘maftools’ package was utilised to generate mutation waterfalls for the 18 genes with the highest mutation frequency in the two risk subgroups.

### Correlation analysis of the CAFRss and the TIME in HCC

The TIMER platform, which uses six algorithms (MCPCOUNTER, TIMER, CIBERSORT, XCELL, QUANTISEQ and EPIC), was used to estimate the level of immune cell infiltration in tumour [21, 22]. Tumour-infiltrating immune cell data were downloaded from the TIMER 2.0 (http://timer.comp-genomics.org/), and Spearman correlation analysis was conducted to determine the determined correlations between risk scores and different levels of immune cell infiltration. The process was implemented and visualised using the ‘ggplot2’, ‘ggtext’, ‘scales’, ‘tidyverse’ and ‘ggpubr’ packages.

Gene set enrichment analysis (GSEA) classifies genomes based on shared biological characteristics [23]. In this study, the single-sample GSEA (ssGSEA) was performed using the packages ‘GSEABase’ and ‘GSVA’ to quantify the degree of immune cell infiltration and immune-related function in each tumour sample from the TCGA-LIHC cohort. The ‘reshape2’ and ‘ggpubr’ packages were then utilised to generate the differential box plots for ssGSEA between the two risk subgroups.

The Estimation of STromal and Immune cells in MAlignant Tumour tissues using Expression data (ESTIMATE) algorithm is commonly employed to estimate the number of infiltrating immune cells and stromal cells in tumour tissues [24]. We utilised the packages ‘ESTIMATE’ and ‘limma’ to estimate the number of immune cells and stromal cells in the tumour tissue of each sample in the TCGA-LIHC cohort to obtain the corresponding scores. Additionally, the R package ‘ggpubr’ was employed to plot differential box plots of immune and stromal scores in the two risk subgroups. Aberrant activation of immune checkpoints inhibits the killing of tumour cells by effector immune cells and promotes immune escape of tumour cells [25]. The differences in the expression of different immune checkpoint-related genes between the two subgroups were analysed.

### Analysis of clinical therapeutic drug sensitivity

The ‘pRRophetic’ package was employed to estimate tumour sensitivity to anti-tumour chemotherapeutic agents at the transcriptome expression level [26]. The IC50 values of different chemotherapeutic agents in different risk subgroups were determined using ‘pRRophetic’ to assess the potential value of CAFRss in guiding clinical pharmacotherapy in patients with advanced HCC [27].

### Statistical analysis

All statistical analyses were processed using the R software (V 4.2.2), and all statistical algorithms were performed by using the corresponding R packages. K-M survival analysis was performed using the log-rank test. *P-*value < 0.05 indicated statistical significance.

## RESULTS

### Identification of CAFs-related genes in HCC

**Figure 2:**
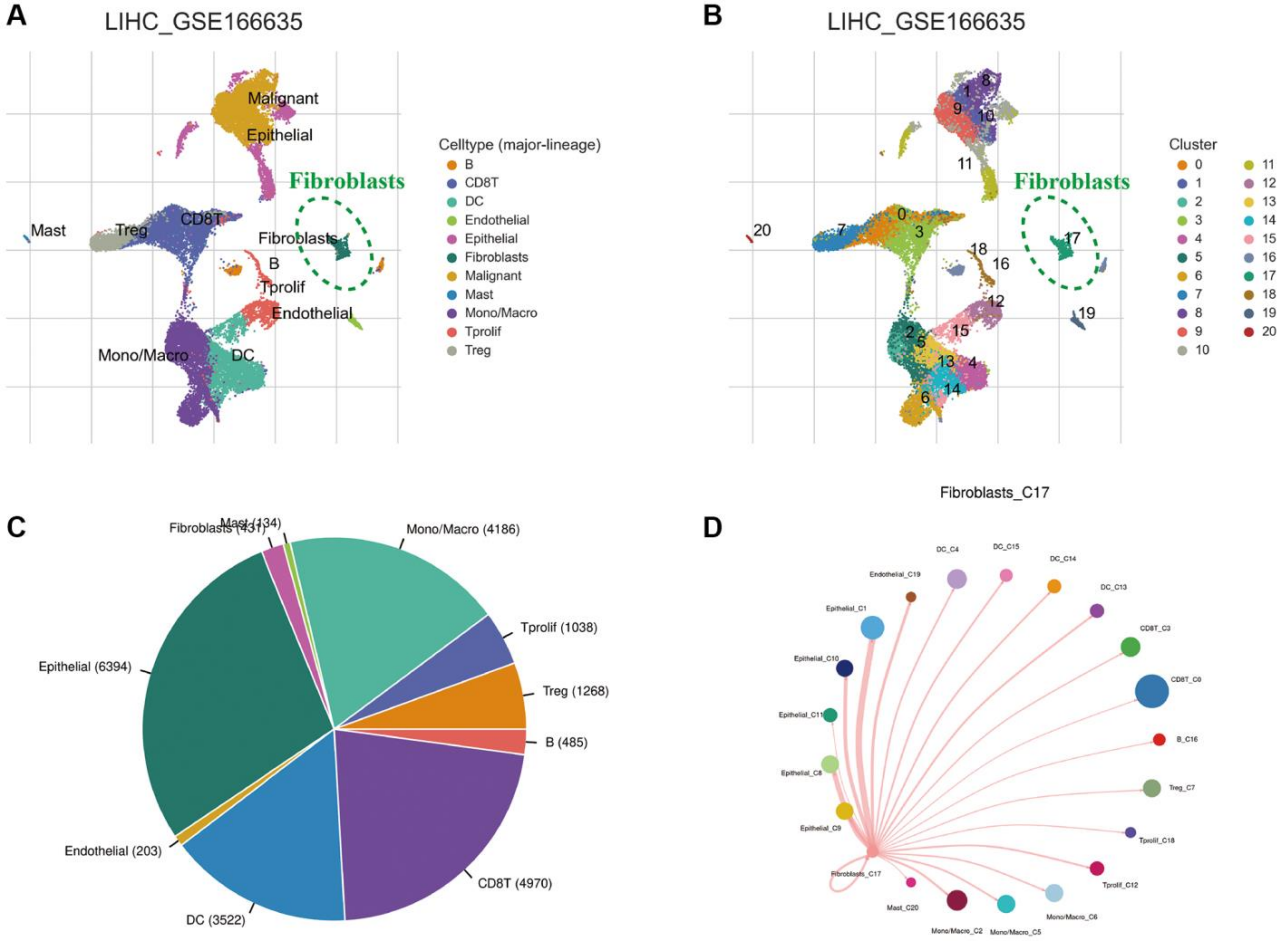
**Single-cell sequencing analysis of HCC tissue.** (**A**) Annotation of cell clusters into 11 cell categories. (**B**) Cluster classification of different cells. (**C**) The proportion of different cell types. (**D**) The communication network between CAFs and other cells.

Cells in the GSE166635 cohort were classified into 11 types based on cell marker annotations in the TISCH database, namely B cells, CD8+ T cells, endothelial cells, fibroblasts, malignant cells, mast cells, dendritic cells, mono-macrophages, epithelial cells, regulatory T cells and proliferating T cells (Figure 2A). Additionally, the T-Distribution Stochastic Neighbour Embedding (tSNE) analysis divided the cell types into 20 clusters (Figure 2B). The pie chart presents the different cell types proportions (Figure 2C). Furthermore, the network diagram reveals the interaction between fibroblasts and other cells (Figure 2D).

**Figure 3:**
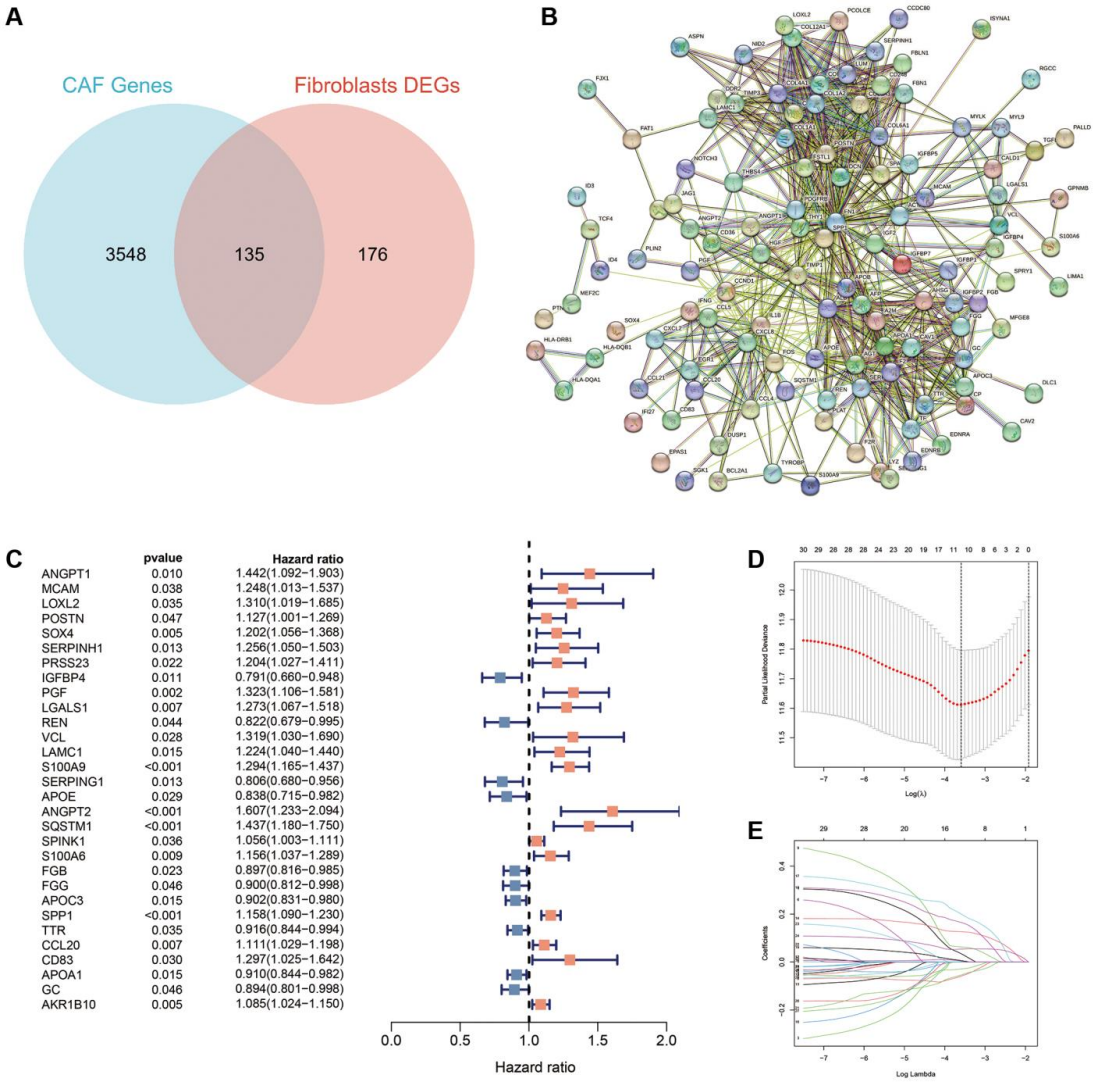
**Identification of CAFs-associated genes in HCC.** (**A**) A total of 311 DEGs and 3683 CAFs-related genes were overlapped to obtain 135 intersecting genes. (**B**) Protein interaction network map of the 135 intersecting genes. (**C**) A total of 30 prognosis-associated genes were obtained using uni-Cox regression. (**D**) The log (lambda) sequence plot of CAFs-related genes using the LASSO algorithm. (**E**) LASSO coefficient profiles of the eleven CAFs-associated genes.

Differential expression analysis identified 311 DEGs between fibroblasts and other cell types. Further intersection with 3683 CAFs-related genes identified 135 DEGs (Figure 3A). The protein interaction network diagram demonstrated the relationships between the intersected genes (Figure 3B).

### Construction of a CAFRss

**Figure 4:**
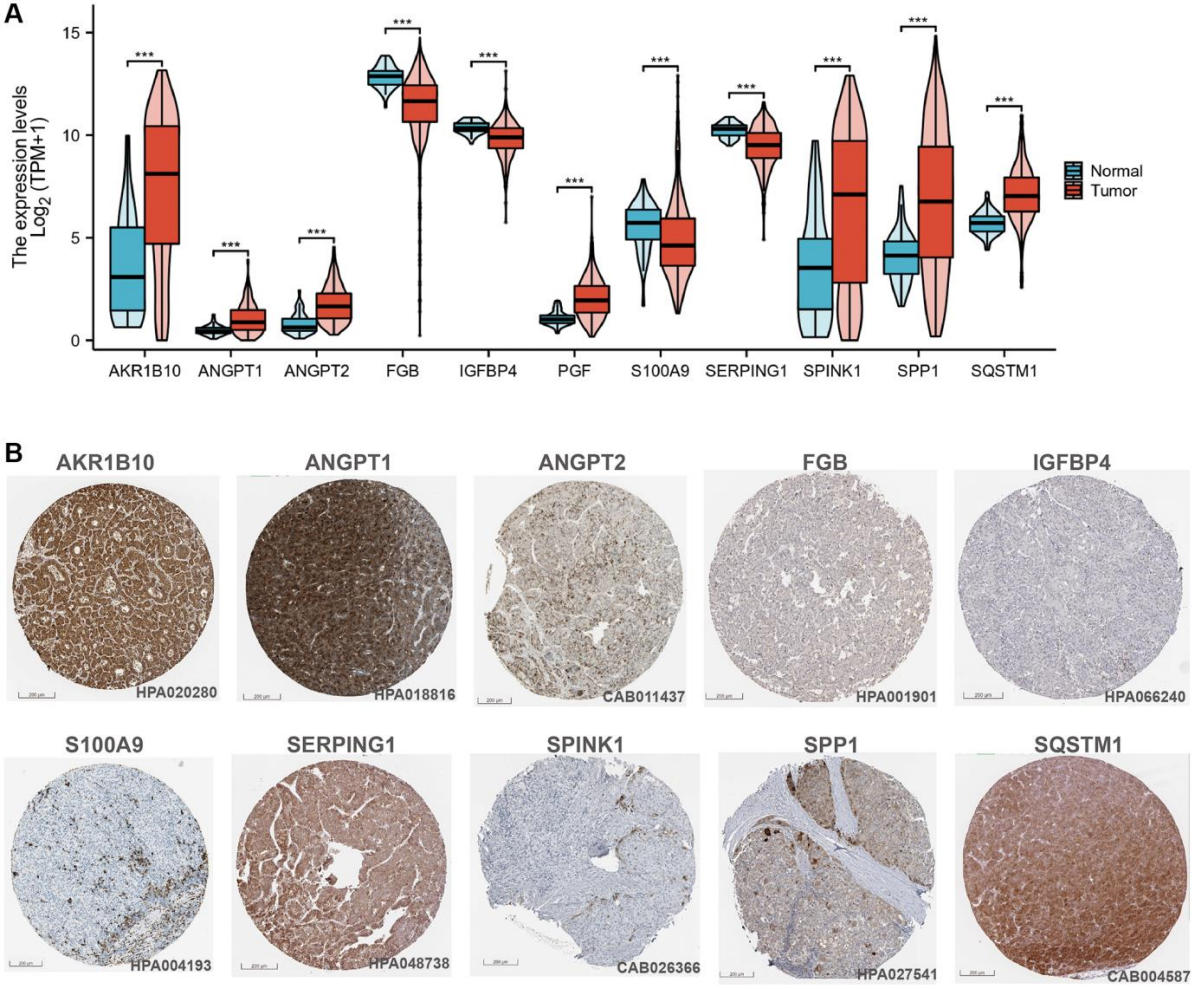
**CAFRss-related genes in HCC.** (**A**) Violin diagram of the expression of CAFRss genes in HCC tumour tissue and normal tissue. (**B**) Immunohistochemical images of proteins encoded by CAFRss-related genes in HCC tumour tissue. ^*^P < 0.05, ^**^P < 0.01, and ^***^P < 0.001.

We performed uni-Cox regression on the 135 CAFs-related genes and identified 30 prognostic genes associated with overall survival (OS), including 10 protective factors and the remaining as risk factors (Figure 3C). To prevent overfitting, the LASSO algorithm further screened 11 of these prognosis-associated genes involved for the establishment of the risk-scoring system (Figure 3D, 3E) (Table 1). Among the 11 CAFRss-related genes, four were downregulated and seven were upregulated in tumour tissues compared to normal tissues (Figure 4A). Additionally, immunohistochemical images from the HPA displayed the protein expression of CAFRss-related genes in HCC tumour tissues (Figure 4B). Furthermore, the expression of the 11 scoring system genes in different cells of HCC tissue is shown in Supplementary Figure 1. Using the risk formula, we obtained a risk score for each sample in the TCGA-LIHC and GSE14520 cohorts. CAFRss (risk score) = *ANGPT1* × (0.03519) − *IGFBP4* × (0.05917) + *PGF* × (0.06325) + *S100A9* × (0.12667) − *SERPING1* × (0.02895) + *ANGPT2* × (0.20089) + *SQSTM1* × (0.16537) + *SPINK1* × (0.00625) − *FGB* × (0.05420) + *SPP1* × (0.05412) + *AKR1B10* × (0.00365). Accordingly, we risk-stratified all patients into high- and low-risk subgroups according to the median risk score of the TCGA-LIHC cohort.

**Table 1:** Risk coefficients for CAFs scoring system-related genes.

**Genes**	**Coefficient**	**HR**	**HR.95L**	**HR.95H**	***p*-value**
ANGPT1	0.03519	1.44180	1.09225	1.90321	0.00980
IGFBP4	−0.05917	0.79059	0.65959	0.94759	0.01101
PGF	0.06325	1.32261	1.10619	1.58136	0.00216
S100A9	0.12667	1.29367	1.16479	1.43682	<0.001
SERPING1	−0.02895	0.80617	0.67980	0.95602	0.01325
ANGPT2	0.20089	1.60705	1.23309	2.09442	<0.001
SQSTM1	0.16537	1.43713	1.18030	1.74983	<0.001
SPINK1	0.00625	1.05565	1.00345	1.11055	0.03633
FGB	−0.05420	0.89696	0.81646	0.98540	0.02342
SPP1	0.05412	1.15773	1.08954	1.23019	<0.001
AKR1B10	0.00365	1.08531	1.02449	1.14975	0.00540

Abbreviations: HR: hazard ratio; CAF: Cancer-associated fibroblasts.

### Validation of the CAFRss

**Figure 5:**
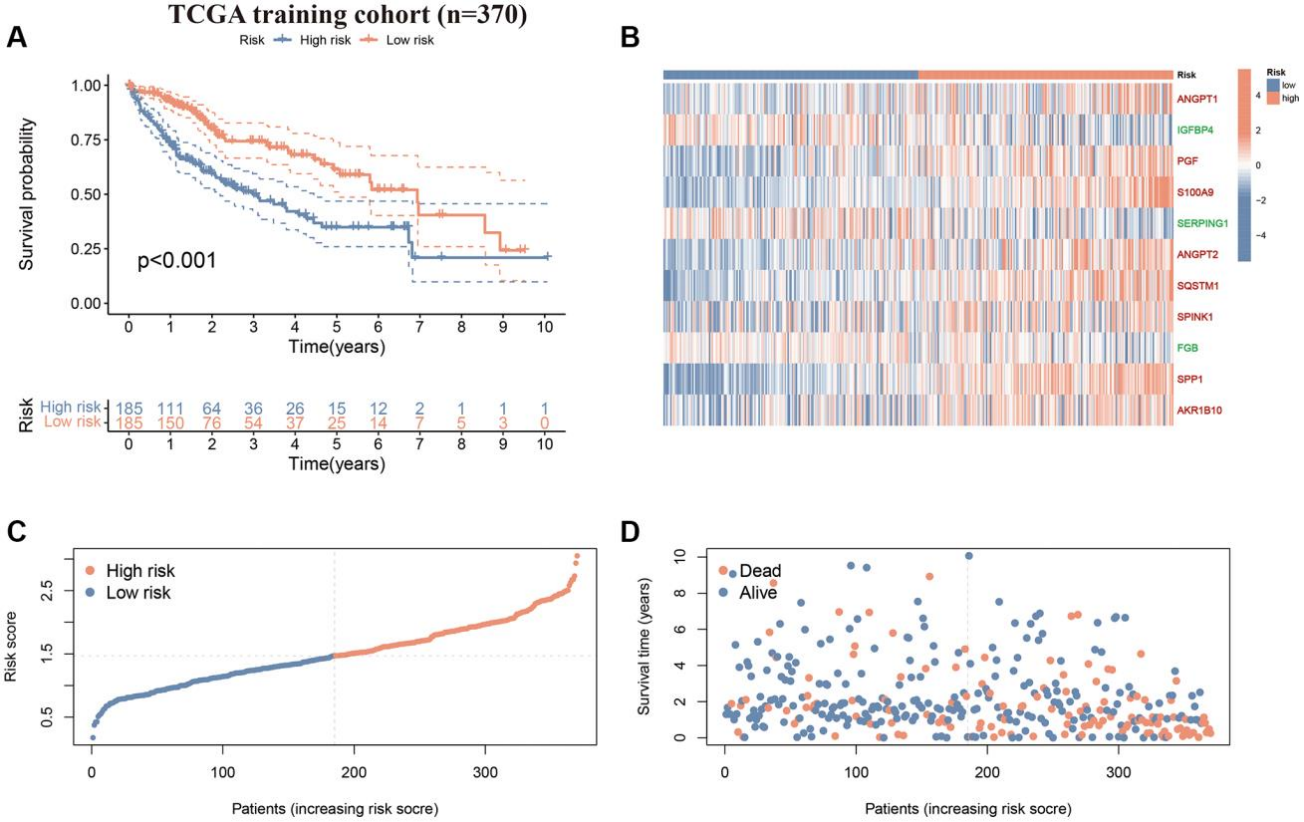
**CAFRss in the TCGA-LIHC cohort.** (**A**) K-M curves for OS. (**B**) Heat map displaying the expression levels of the 11 genes in the CAFRss (Red genes represent risk factors; green genes represent protective factors). (**C**, **D**) Risk score distribution curves and survival status plots.

**Figure 6:**
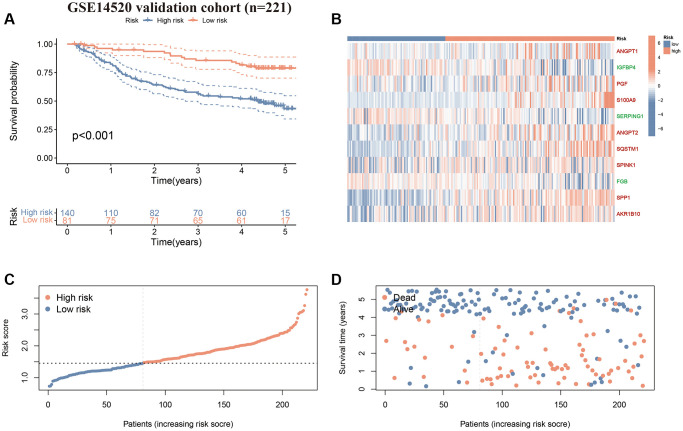
**Validation of the CAFRss in GSE14520 cohort.** (**A**) K-M curves for OS. (**B**) Heat map displaying the expression levels of the 11 genes in CAFRss (Red genes represent risk factors; green genes represent protective factors). (**C**, **D**) Risk score distribution curves and survival status plots.

First, we performed survival validation of the CAFRss in the TCGA-LIHC cohort. The K-M curves demonstrated that the low-risk population had significantly better OS than the high-risk subgroup (Figure 5A). The heat map displayed the expression levels of the 11 genes in the CAFRss, where the three protective factors (*IGFBP4*, *SERPING1* and *FGB*) exhibited lower expression in the high-risk subgroup, while the eight risk factors exhibited higher levels of expression in the high-risk subgroup (Figure 5B). Furthermore, the risk score distribution and survival status maps for the TCGA-LIHC cohort showed an increasing proportion of individuals with mortality status as the high-risk scores rose (Figure 5C, 5D). The results were further validated in the GSE14520 cohort (Figure 6A–6D).

### Evaluation of the CAFRss

**Figure 7:**
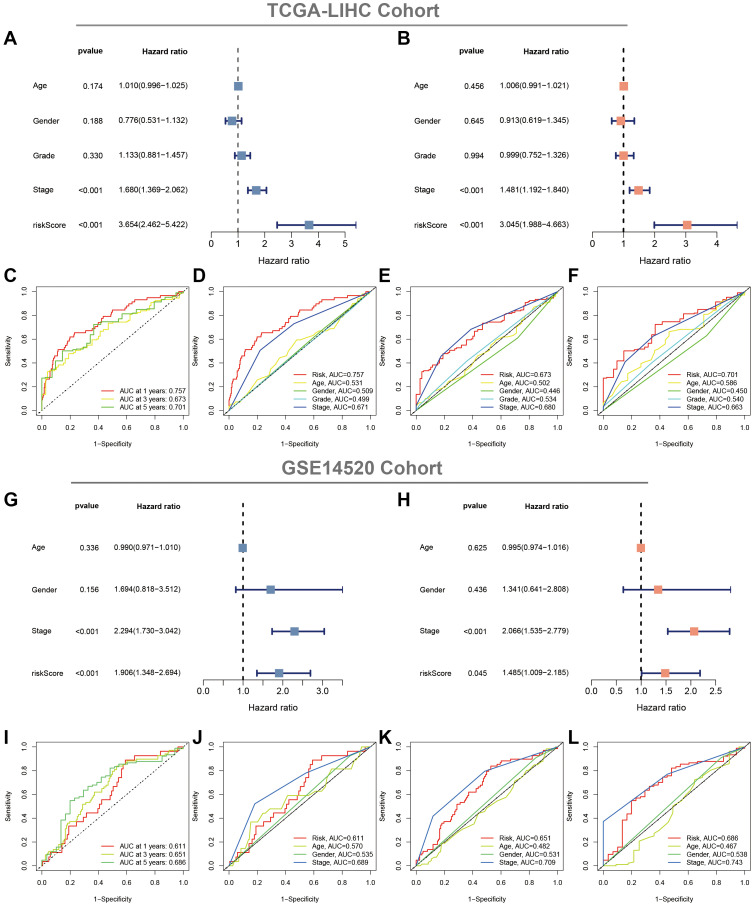
**Evaluation of the CAFRss.** (**A**, **B**) Forest plots of uni- and multi-Cox analyses of the TCGA cohort. (**C**) ROC curves for the CAFRss. (**D**–**F**) Comparison of AUC of CAFRss with age, stage, grade and gender at 1, 3 and 5 years. (**G**, **H**) Forest plots of uni- and multi-Cox analyses of the GSE14520 cohort. (**I**) ROC curves for the CAFRss. (**J**–**L**) Comparison of AUC of CAFRss with age, stage and gender at 1-, 3- and 5-years in the GSE14520 cohort.

Uni- and multi-Cox regression analyses indicated that CAFRss was an independent prognostic indicator for patients with HCC, with hazard ratios of 3.654 (*P* < 0.001) and 3.045 (*P* < 0.001) (Figure 7A, 7B). Moreover, the stage was also identified as an independent prognostic indicator (*P* < 0.001). Additionally, ROC curves were performed to assess the predictive efficacy of CAFRss in predicting survival in the TCGA-LIHC cohort. The area under the curve (AUC) values for CAFRss in predicting OS at 1, 3 and 5 years were 0.757, 0.673 and 0.701, respectively (Figure 7C–7F). Furthermore, the CAFRss was also confirmed as an independent prognostic factor in the GSE14520 cohort (Figure 7G, 7H), with AUC values of 0.611, 0.651 and 0.686 at 1, 3 and 5 years (Figure 7I–7L).

### Nomogram for HCC

**Figure 8:**
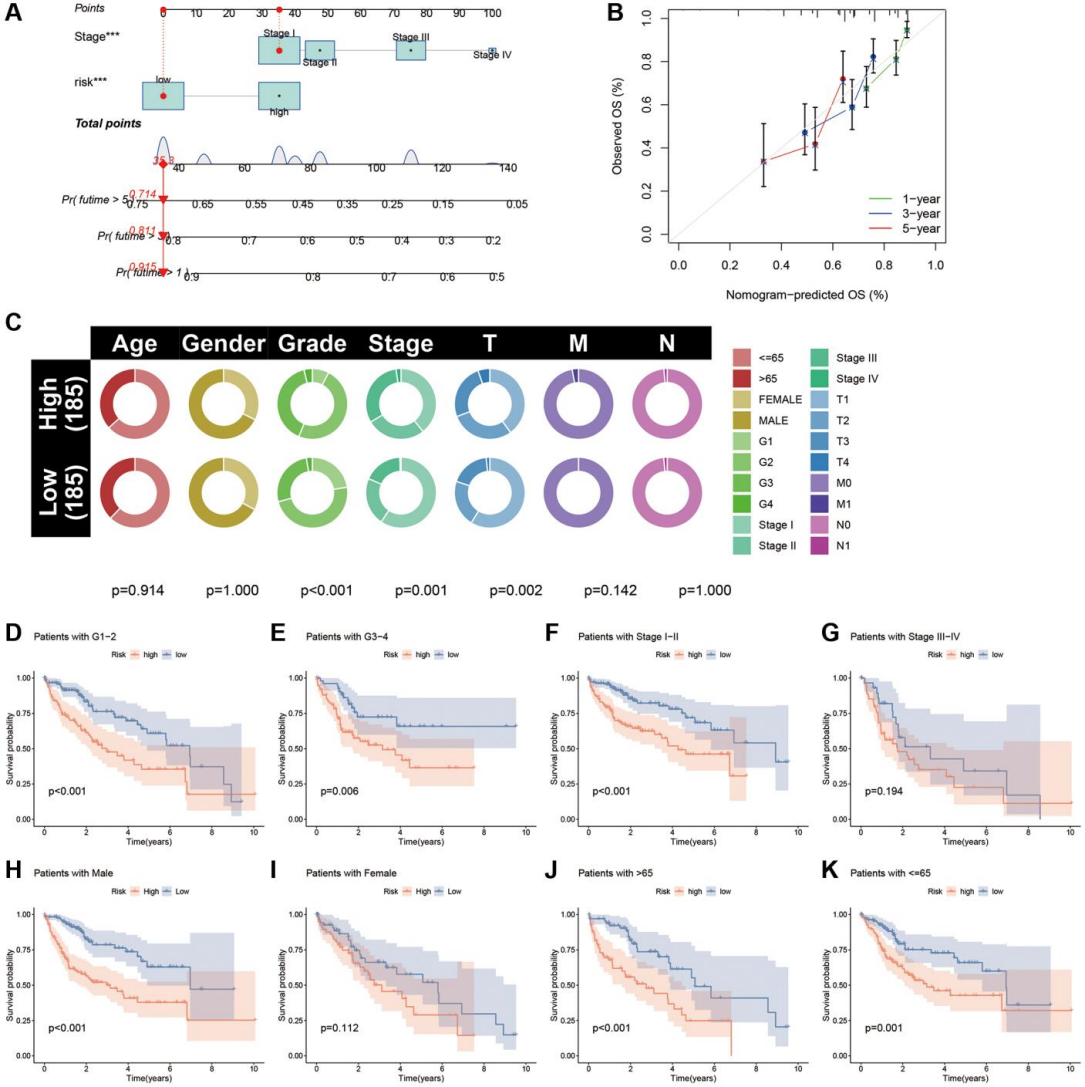
**Nomogram and clinical subgroup analysis based on CAFRss.** (**A**) Construction of a nomogram for HCC. (**B**) The calibration curves for the nomogram. (**C**) Circle plots show the differences in clinical parameters between the two risk subgroups. (**D**–**K**) The K-M curves show the survival differences between the high- and low-risk subgroups in the tumour grade (**D**, **E**), tumour stage (**F**, **G**), gender (**H**, **I**) and age (**J**, **K**) subgroups.

Based on the multi-Cox regression results, we incorporated the CAFRss and TNM staging into the construction of the nomogram to predict the OS of patients with HCC at 1, 3 and 5 years (Figure 8A). Moreover, the calibration curves showed that the nomogram’s predictions for HCC survival were in good agreement with the actual outcomes (Figure 8B).

### Stratified analysis of clinicopathological parameters

Circle plots of the clinical parameters revealed significant differences in grade, tumour stage and T stage between the two risk subgroups (Figure 8C). To assess the applicability of CAFRss in patients with different clinical subtypes, we performed a stratified analysis based on different clinicopathological parameters and further validated the survival in different subgroups. K-M curves demonstrated that individuals with different tumour grade and age had a better survival in the low-risk subgroup (*P* < 0.05) (Figure 8D–8K). Although there is no significant difference in survival between risk subgroups for women and stage III–IV patients, a noticeable trend towards the separation of the K-M curves was observed.

### GSVA, GO and KEGG analysis

**Figure 9:**
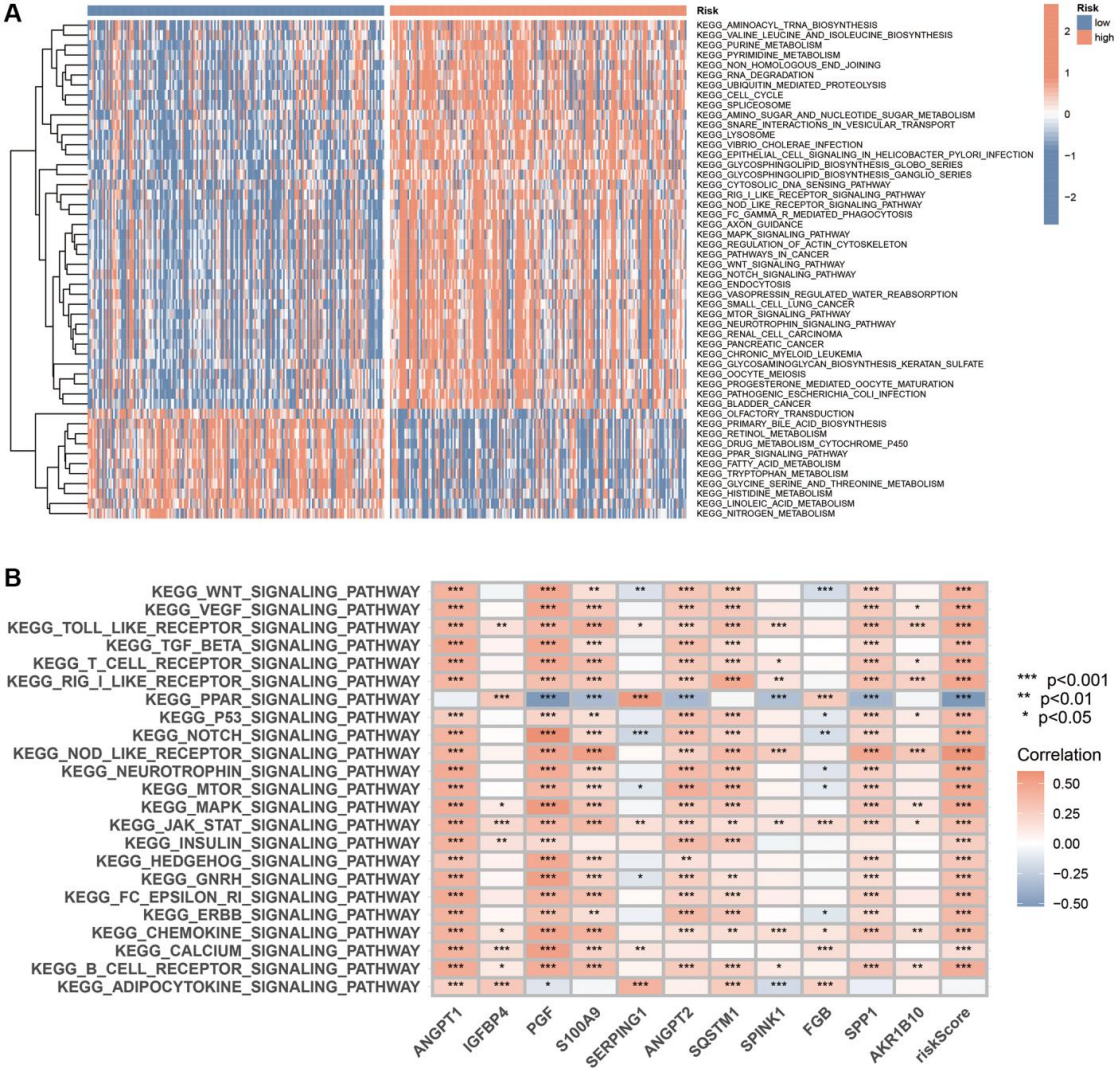
**GSVA analysis based on the CAFRss.** (**A**) The heat map displays the KEGG pathways enriched in the two risk subgroups. (**B**) The heat map demonstrates the correlation between the expression of 11 genes in the CAFRss and key tumour-related signalling pathways. ^*^P < 0.05, ^**^P < 0.01, and ^***^P < 0.001.

We first utilised GSVA to identify the pathways enriched in the different risk subgroups. The analysis revealed that the pathways enrichment of the MAPK, WNT, Notch, NOD-like receptor, RIG I-like receptor and mTOR signalling pathways, which are extensively involved in tumour evolution, in the high-risk subgroup. Additionally, the PPAR signalling pathway and multiple metabolic pathways were enriched in the low-risk subgroup (Figure 9A). Furthermore, a significant correlation was observed between the expression of 11 genes in the scoring system and key signalling pathways (Figure 9B).

**Figure 10:**
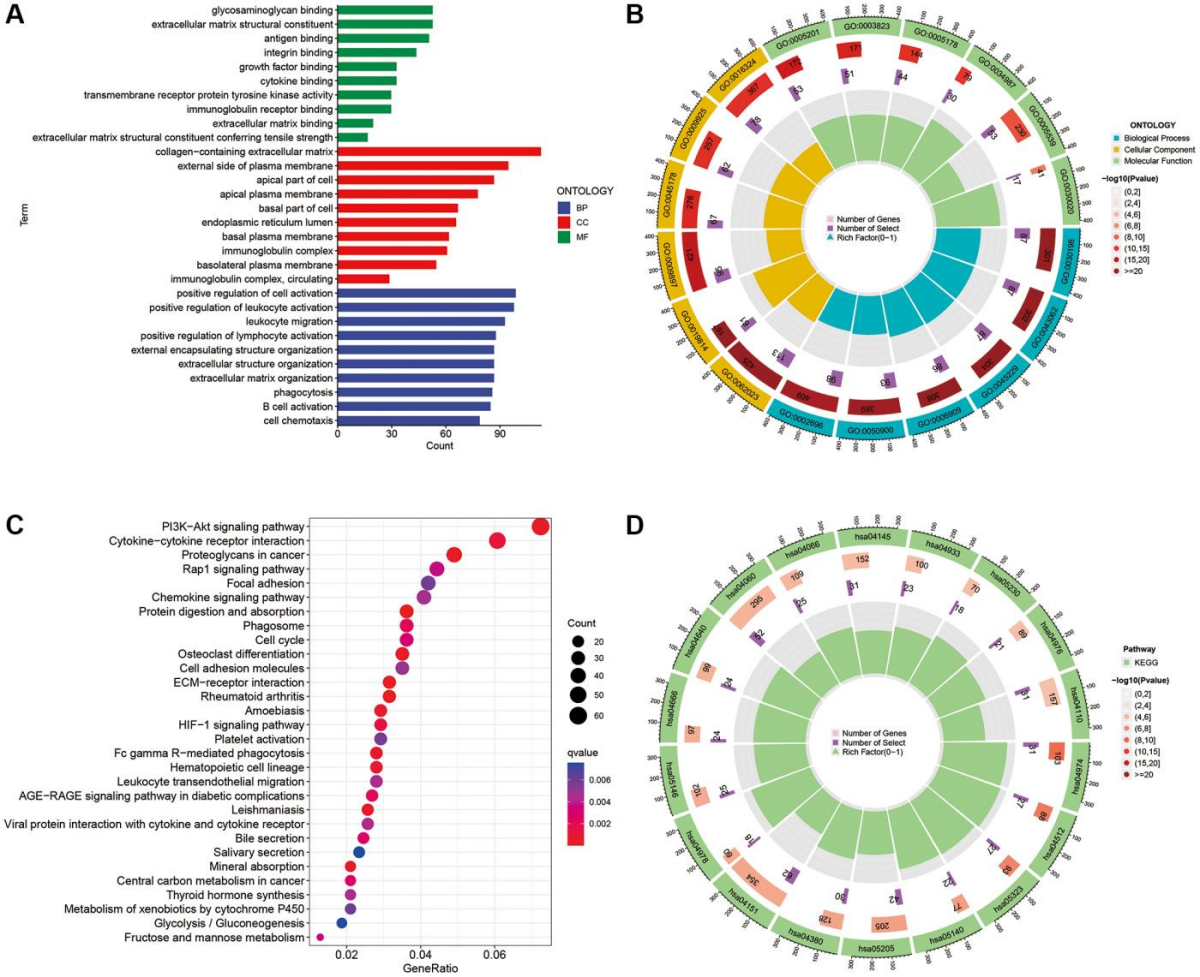
**GO and KEGG enrichment analysis.** (**A**, **B**) GO analysis of DEGs between the two risk subgroups. (**C**, **D**) KEGG analysis of the DEGs between the two risk subgroups.

Considering the differences in the enrichment pathways between the different risk subgroups, we identified DEGs between the two risk subgroups and further performed GO and KEGG analyses. GO analysis indicated that the DEGs were mainly enriched in the positive regulation of cell activation, leukocyte activation, leukocyte migration, lymphocyte activation, collagen-containing extracellular matrix, immunoglobulin complex and antigen binding, among others (Figure 10A, 10B). Regarding KEGG, DEGs were mainly enriched in PI3K-Akt signalling pathway, cytokine-cytokine receptor interactions, proteoglycans in cancer, Rap1 signalling pathway, focal adhesion and chemokine signalling pathways (Figure 10C, 10D).

### Correlation of the CAFRss and TMB

**Figure 11:**
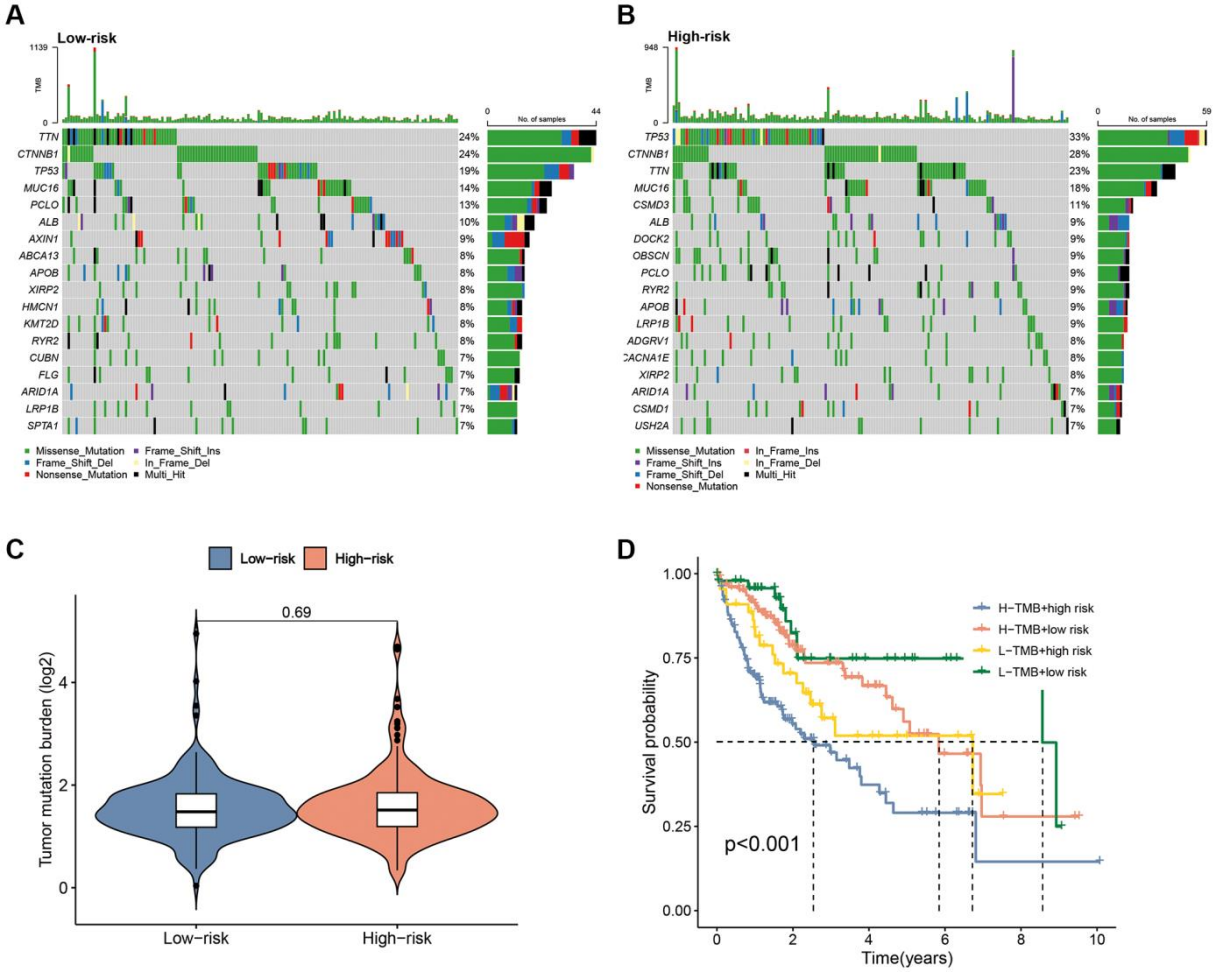
**Correlation of the CAFRss with TMB.** (**A**, **B**) Mutation waterfall plots in the two subgroups. (**C**) Comparison of TMB between the two risk subgroups. (**D**) K-M curves for the TMB subgroups combined with the risk subgroups.

Mutation waterfall plots revealed the top three genes with the highest mutation frequencies in the low-risk subgroup as *CTNNB1* (24%), *TTN* (24%) and *TP53* (19%), whereas, in the high-risk group it was *TP53* (33%), *CTNNB1* (28%) and *TTN* (23%) (Figure 11A, 11B). K-M curves demonstrated that patients with low TMB had significantly better survival compared to those with high-TMB (Supplementary Figure 2). Although there was no significant difference in TMB levels between the two risk subgroups (Figure 11C), there was a significant difference in survival between the TMB subgroup and the risk subgroup combination (*P* < 0.001) (Figure 11D), suggesting that the combination of risk score and TMB could provide a better prediction of clinical outcomes for patients.

### Correlation of the CAFRss with TIME

**Figure 12:**
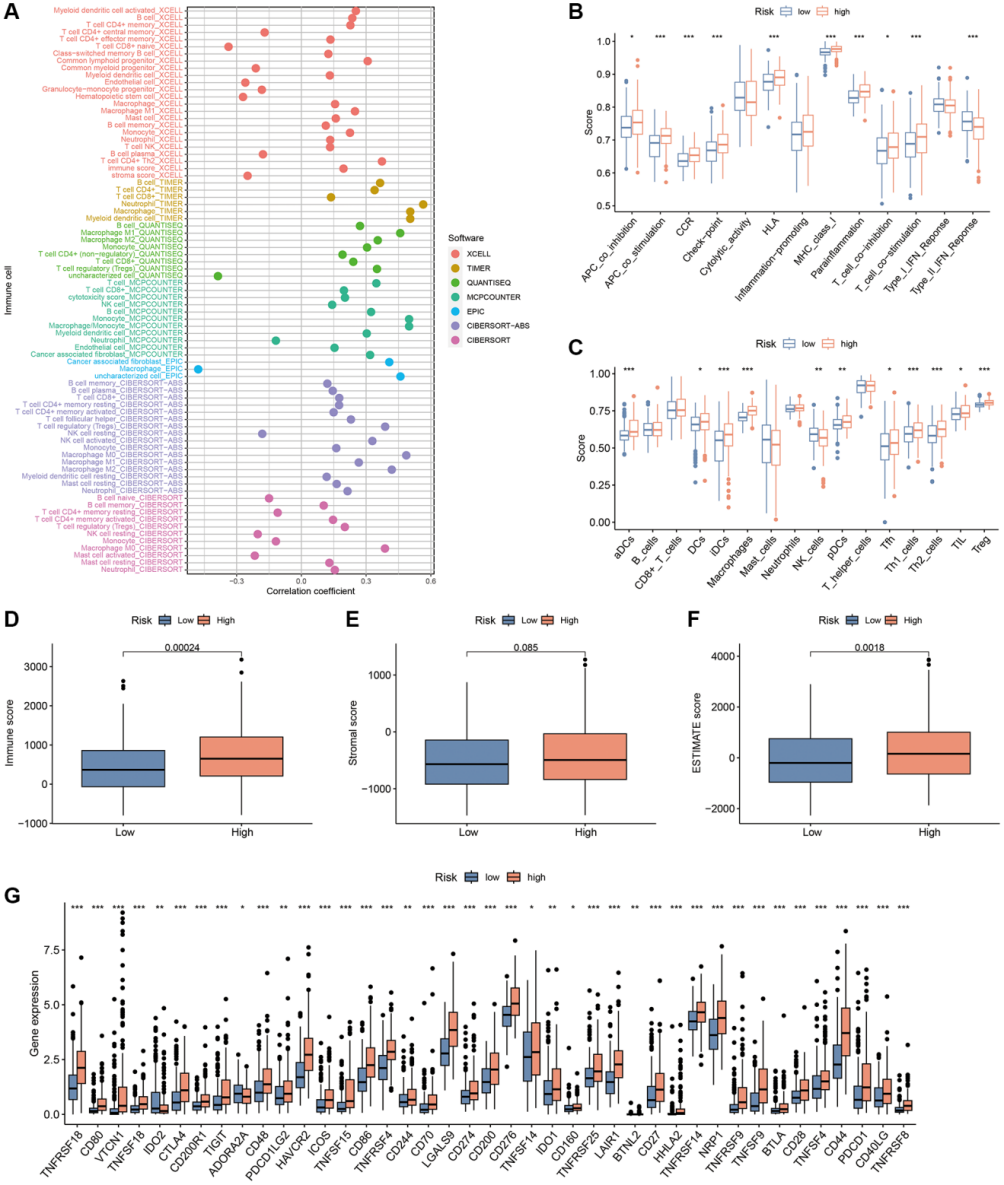
**TIME analysis based on the CAFRss.** (**A**) Bubble plots display the correlation between risk scores and different immune cells in the TIMER 2.0 platform. (**B**) ssGSEA shows differences in immune function score across the subgroups. (**C**) ssGSEA shows differences in immune cells score across the risk groups. (**D**–**F**) Box plots display the differences in immune, stromal and ESTIMATE scores across the two subgroups. (**G**) Box plots show the differences in expression of immune checkpoints across the risk subgroups. ^*^P < 0.05, ^**^P < 0.01, and ^***^P < 0.001.

Considering the enrichment of immune-associated functions in GO analysis, we further explored the relationship between CAFRss and the TIME in HCC. TIMER 2.0 analysis indicated that the degree of infiltration of most immune cells was positively correlated with risk scores (Figure 12A). Additionally, ssGSEA revealed that immune-related functions such as APC co-stimulation and co-inhibition, chemokine receptors, immune checkpoints, human leukocyte antigens, MHC class I, para-inflammation, T cell co-stimulation and co-inhibition and type II IFN response were significantly higher in the high-risk subgroup compared to the low-risk subgroup (Figure 12B). In terms of immune cells, the high-risk subgroup showed significantly higher levels of dendritic cells, activated dendritic cells, immature dendritic cells, macrophages, follicular helper T cells, helper T cells, tumour-infiltrating lymphocytes and regulatory T cells (Figure 12C). Furthermore, ESTIMATE analysis revealed higher ESTIMATE scores and immune scores in the high-risk subgroup, while stromal scores did not differ significantly between the two subgroups (Figure 12D–12F).

ICBs therapy mainly involves the blockade of immune checkpoints, thereby restoring the immune system’s ability to recognise tumour cells. The analysis of immune checkpoints, including Programmed Cell Death Protein 1 (PD1, encoded by PDCD1), Programmed Cell Death-Ligand 1 (PD-L1, encoded by CD274) and cytotoxic T-lymphocyte associated antigen 4 (CTLA-4), indicated higher expression levels in the high-risk group (Figure 12G), suggesting that the high-risk group may be a beneficial population for ICBs treatment.

### The predictive value of CAFRss for anti-tumour drug sensitivity

**Figure 13:**
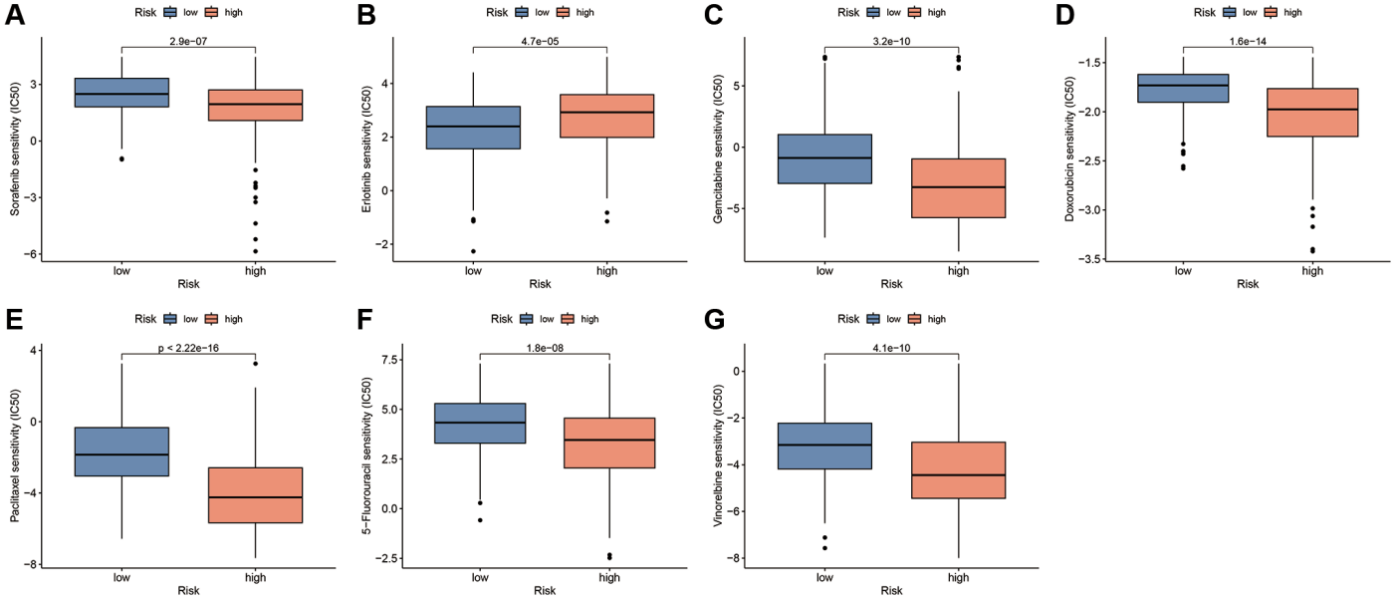
**Drug sensitivity analysis based on the CAFRss.** (**A**–**G**) Box plots display the drugs with different IC50 values across the risk subgroups.

To explore the potential of CAFRss in the personalised treatment of patients with HCC, we analysed the differences in IC50 values for different anti-tumour agents between the two risk subgroups. Notably, with the exception of erlotinib, the IC50 for sorafenib and most chemotherapeutic agents was lower in the high-risk group compared to the low-risk subgroup (Figure 13A–13G). Additionally, the IC50s for the targeted drugs dasatinib, imatinib, lisitinib, sunitinib and ruxolitinib were also significantly lower in the high-risk subgroup (Supplementary Figure 3).

## DISCUSSION

Tumour development is influenced by both intrinsic properties and the specific tumour microenvironment (TME) [28]. The different components of the TME play an important role in tumour growth, invasion, metastasis, angiogenesis, immunosuppression and drug resistance [29–31]. Among the components of the TME, CAFs play a crucial role. CAFs interact extensively with tumour cells throughout tumourigenesis and tumour development, affecting tumour growth, metastasis, invasion, treatment resistance and immunosuppression [32–34]. There is increasing evidence linking CAFs to the effectiveness of tumor immunotherapy, making them a potential target for improving immunotherapy outcomes [19, 35–37]. Previous studies have demonstrated the impact of CAFs on the biological behaviour of tumours and their involvement in targeted therapy and immunotherapy resistance [16, 38, 39]. Thus, understanding the unique function of CAFs in assessing the clinical outcomes and the efficacy of immunotherapy in patients with HCC will aid in the development of more effective predictive biomarkers and molecular targets.

Interdisciplinary analyses based on large amounts of sequencing data and diverse bioinformatics algorithms are increasingly being used for the identification of tumor subtypes, providing a valuable reference in the discovery of potential biomarkers [40–43]. In this study, a combined analysis of single-cells and bulk RNA sequencing was performed to construct a CAFRss comprising 11 CAFs-related genes. The CAFRss was developed using Cox and LASSO algorithms to predict clinical outcome in patients with HCC. The prognostic predictive value of the CAFRss was validated in independent cohort GSE14520. Cox regression further confirmed CAFRss as an independent prognostic factor in HCC. Additionally, ROC curves indicated superior prognostic predictive efficacy of the CAFRss, indicating its reliability as a prognostic prediction tool for patients with HCC.

Tumourigenesis induces and establishes a TIME that favours immunosuppression, leading to the loss of anti-tumour function of effector immune cells and the activation of immunosuppressive molecules, thus triggering immune escape. Immune checkpoints (ICs) are important regulators of this process, as ICs can signal ‘immune brakes’ to suppress effector immune function, and their dysregulation can contribute to immune escape in tumours [44, 45]. Recently, ICBs, the classical representatives of immunotherapy, have revolutionised the treatment landscape for solid tumours [46]. Despite the promising potential of ICBs, their overall inefficiency is a pressing issue in clinical immunotherapy. Thus, there is an urgent need to explore reliable predictive biomarkers that can identify populations that would benefit from treatment with ICBs, thereby aiding clinical decision-making and enhancing individualised treatment regimens.

Recent studies have confirmed that low levels of infiltration of effector immune cells in tumour tissues, also known as immune ‘cold tumour’, are speculated to be the main contributors to the low response rate of ICBs [47]. Conversely, immune ‘hot tumours’ have a better response rate to ICBs, featuring the activation of immune checkpoints and a high infiltration level of immune cells [48, 49]. Notably, most of the immune checkpoints including PD1, PD-L1 and CTLA-4 were significantly higher in the high-risk subgroup than in the low-risk population for CAFRss, indicating a higher state of immunosuppression in the high-risk subgroup, which may partly explain the poorer prognosis in this population. Additionally, the systematic analysis of the TIMER 2.0, ssGSEA and ESTIMATE algorithms demonstrated a higher degree of immune infiltration in the high-risk subgroup, suggesting that the high-risk population in the CAFRss is more consistent with an immune ‘hot tumour’ profile. These findings imply that the high-risk population based on CAFRss may benefit more from treatment with ICBs compared to low-risk populations.

Targeted therapies, especially tyrosine kinase inhibitors (TKIs), are a mainstay of systemic therapy for advanced HCC. Based on the results of the SHARP and ORIENTAL studies, sorafenib was identified as the first multi-targeted small molecule TKI and approved for unresectable HCC. The median survival time was prolonged by 2.8 months in the sorafenib group compared to the placebo group [50]. In this study, patients in the high-risk subgroup showed greater sensitivity to sorafenib, suggesting that this subgroup may benefit more from sorafenib treatment. Additionally, a meta-analysis showed that the TKI erlotinib was effective in combination with bevacizumab in patients with sorafenib-resistant HCC [51]. In the present study, low-risk patients were observed to benefit more from erlotinib treatment. Notably, chemotherapeutic agents such as 5-fluorouracil, doxorubicin, gemcitabine, paclitaxel and vinorelbine had lower IC50 values in the low-risk subgroup, suggesting potential chemotherapy resistance in the low-risk group.

Despite the comprehensive methodological evaluations and validations of the developed CAFRss in this study, certain limitations should be acknowledged. Firstly, this study was unable to examine the potential biases of the data included in this retrospective study. Secondly, the clinical value of CAFRss in HCC remains to be further validated in a prospective clinical trial with a large sample size.

## CONCLUSION

The CAFRss developed in this study show superior predictive ability for the clinical outcome of patients with HCC compared to traditional clinicopathological parameters. Moreover, CAFRss-based population stratification could effectively differentiate between ‘hot’ and ‘cold’ immune tumours, providing a basis for the application of ICBs and the selection of personalised therapeutic plans for patients with HCC.

## Supplementary Materials

Supplementary Figures

Supplementary Table 1
