# Supplementary material for: Identification and validation of a cancer-associated fibroblasts-related scoring system to predict prognosis and immune landscape in hepatocellular carcinoma through integrated analysis of single-cell and bulk RNA-sequencing
Source: Aging (Albany NY). 2023 Oct 18;15(20):11092–113. doi: 10.18632/aging.205099 (PMC10637792; doi:10.18632/aging.205099)
Supplement: Supplementary Figures [file aging-15-205099-s001.pdf]

SUPPLEMENTARY FIGURES

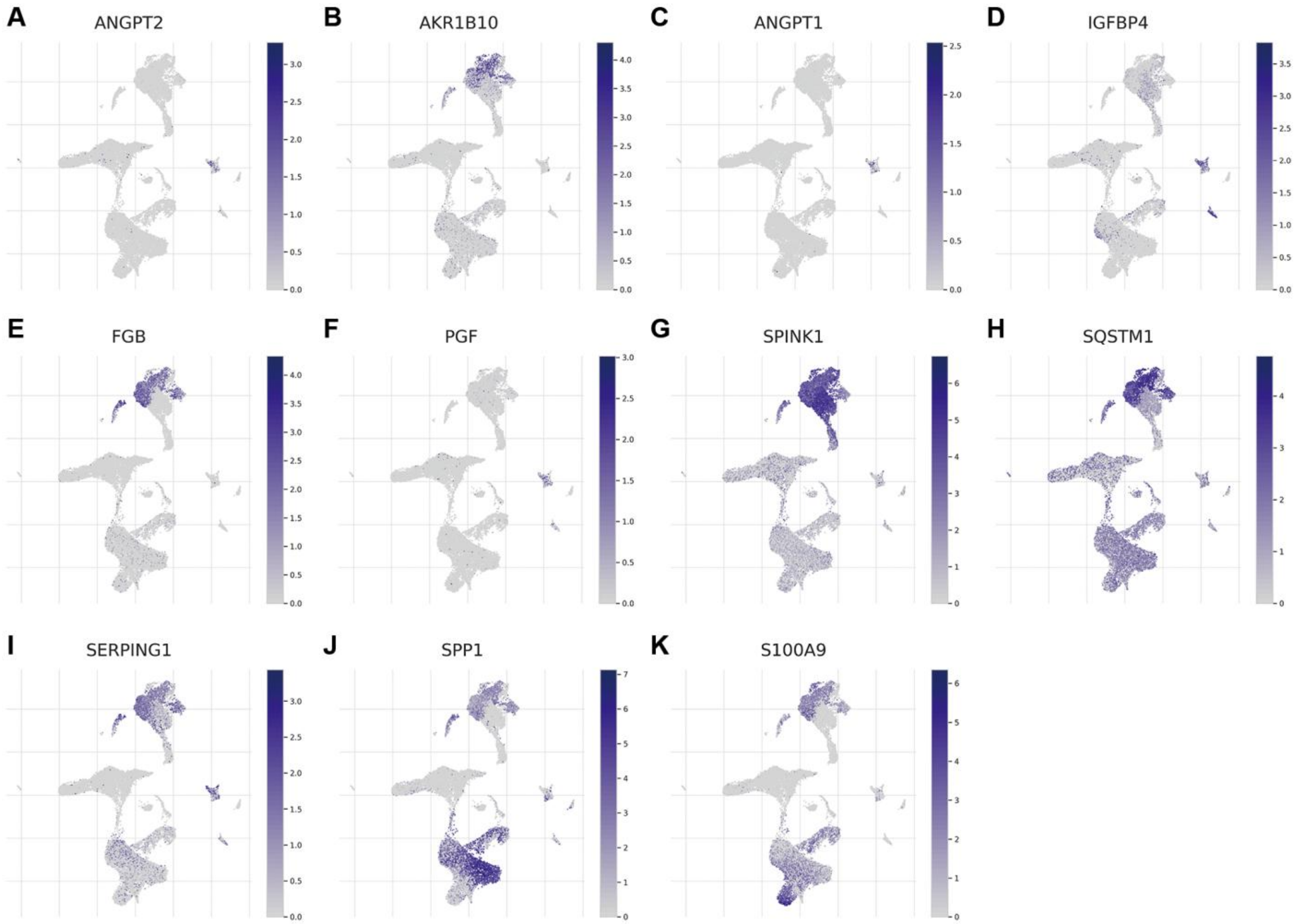

Supplementary Figure 1. Single-cell sequencing analysis of CAFRss-related genes. (**A**–**K**) The expression of the 11 scoring system-related genes in the different cell types of HCC tissue.

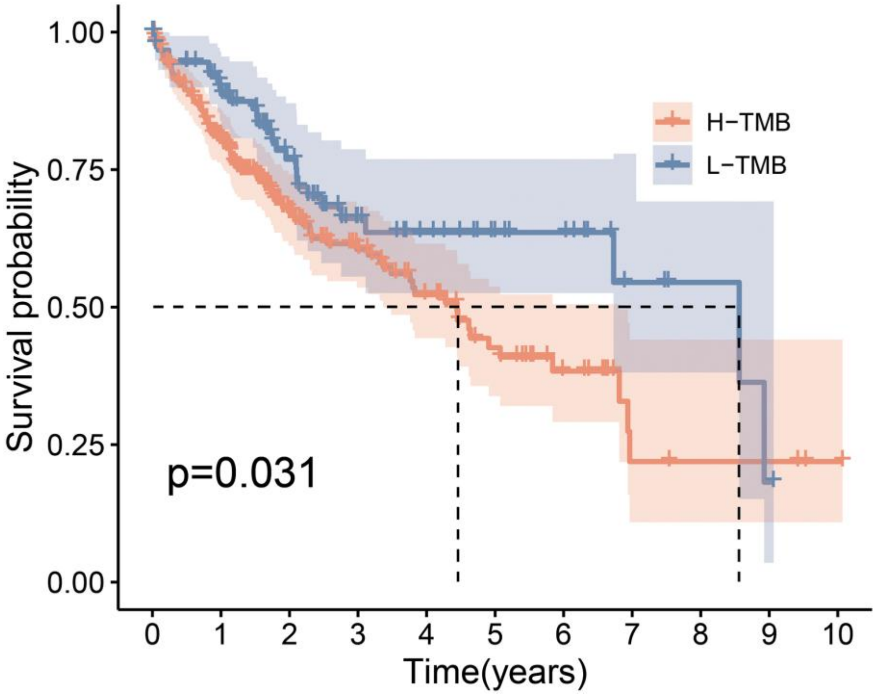

Supplementary Figure 2. Kaplan Meier curves of the high- and low-TMB groups in the TCGA-LIHC cohort.

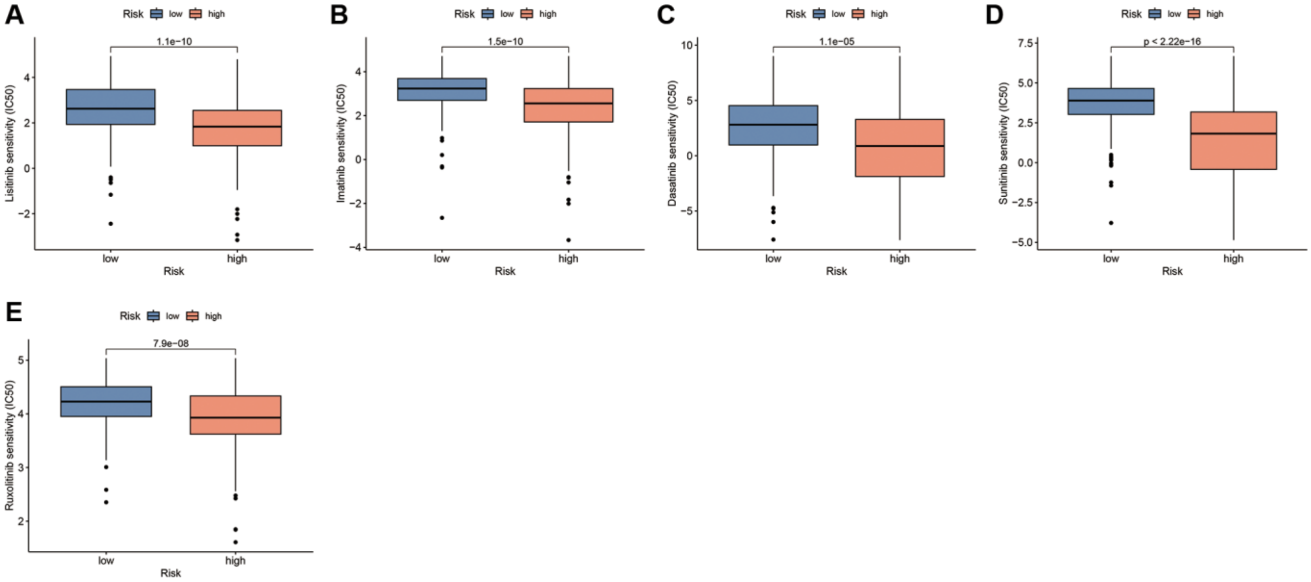

Supplementary Figure 3. Drug sensitivity analysis based on the CAFRss. (**A**–**E**) Box plots display the targeted drugs with different IC50 values between the risk groups.
