# Supplementary material for: Identification and validation of a cancer-associated fibroblasts-related scoring system to predict prognosis and immune landscape in hepatocellular carcinoma through integrated analysis of single-cell and bulk RNA-sequencing
Source: Aging (Albany NY). 2023 Oct 18;15(20):11092–113. doi: 10.18632/aging.205099 (PMC10637792; doi:10.18632/aging.205099)
Supplement: Supplementary Table 1 [file aging-15-205099-s002.pdf]

SUPPLEMENTARY TABLE

Supplementary Table 1. IHC staining images of proteins encoded by CAFRss-related genes in HPA.

CAFRss-related genes	Human protein atlas
ANGPT1	https://www.proteinatlas.org/ENSG00000154188-ANGPT1/pathology/liver+cancer
IGFBP4	https://www.proteinatlas.org/ENSG00000141753-IGFBP4/pathology/liver+cancer
S100A9	https://www.proteinatlas.org/ENSG00000163220-S100A9/pathology/liver+cancer
SERPING1	https://www.proteinatlas.org/ENSG00000149131-SERPING1/pathology/liver+cancer
ANGPT2	https://www.proteinatlas.org/ENSG00000091879-ANGPT2/pathology/liver+cancer
SQSTM1	https://www.proteinatlas.org/ENSG00000161011-SQSTM1/pathology/liver+cancer
SPINK1	https://www.proteinatlas.org/ENSG00000164266-SPINK1/pathology/liver+cancer
FGB	https://www.proteinatlas.org/ENSG00000171564-FGB/pathology/liver+cancer
SPP1	https://www.proteinatlas.org/ENSG00000118785-SPP1/pathology/liver+cancer
AKR1B10	https://www.proteinatlas.org/ENSG00000198074-AKR1B10/pathology/liver+cancer
